# Iron-Oxide Minerals Affect Extracellular Electron-Transfer Paths of *Geobacter* spp

**DOI:** 10.1264/jsme2.ME12161

**Published:** 2013-01-30

**Authors:** Souichiro Kato, Kazuhito Hashimoto, Kazuya Watanabe

**Affiliations:** 1 Hashimoto Light Energy Conversion Project, ERATO, JST, 7–3–1 Hongo, Bunkyo-ku, Tokyo, 113–8656, Japan; 2 Department of Applied Chemistry, The University of Tokyo, 7–3–1 Hongo, Bunkyo-ku, Tokyo, 113–8656, Japan; 3 Research Center for Advanced Science and Technology, The University of Tokyo, 4–6–1 Komaba, Meguro-ku, Tokyo, 153–8904, Japan; 4 School of Life Sciences, Tokyo University of Pharmacy and Life Sciences, 1432–1 Horinouchi, Hachioji, Tokyo 192–0392, Japan

**Keywords:** extracellular electron transfer, energy metabolism, microbial fuel cell, *Geobacter*, iron oxide

## Abstract

Some bacteria utilize (semi)conductive iron-oxide minerals as conduits for extracellular electron transfer (EET) to distant, insoluble electron acceptors. A previous study demonstrated that microbe/mineral conductive networks are constructed in soil ecosystems, in which *Geobacter* spp. share dominant populations. In order to examine how (semi)conductive iron-oxide minerals affect EET paths of *Geobacter* spp., the present study grew five representative *Geobacter* strains on electrodes as the sole electron acceptors in the absence or presence of (semi)conductive iron oxides. It was found that iron-oxide minerals enhanced current generation by three *Geobacter* strains, while no effect was observed in another strain. *Geobacter sulfurreducens* was the only strain that generated substantial amounts of currents both in the presence and absence of the iron oxides. Microscopic, electrochemical and transcriptomic analyses of *G. sulfurreducens* disclosed that this strain constructed two distinct types of EET path; in the absence of iron-oxide minerals, bacterial biofilms rich in extracellular polymeric substances were constructed, while composite networks made of mineral particles and microbial cells (without polymeric substances) were developed in the presence of iron oxides. It was also found that uncharacterized *c*-type cytochromes were up-regulated in the presence of iron oxides that were different from those found in conductive biofilms. These results suggest the possibility that natural (semi)conductive minerals confer energetic and ecological advantages on *Geobacter*, facilitating their growth and survival in the natural environment.

Some dissimilatory metal-reducing bacteria (DMRBs) are able to utilize extracellular insoluble electron acceptors (*e.g.*, metal oxides and electrodes) for their respiration (7, 19, 49). This is termed extracellular electron transfer (EET) and recognized as important energy-conservation processes in natural and engineered environments (*e.g.*, global carbon/metal cycling, bioremediation of toxic metals, and microbial fuel/electrolysis cells) (18–21, 55, 57).

Three distinct mechanisms for microbial EET have previously been proposed (7, 21, 51). The first mechanism is direct electron transfer (ET) mediated by outer-membrane (OM) *c*-type cytochromes (*c*-Cyts) (17, 25, 33, 38, 45). Microorganisms using this mechanism have to directly attach to solid materials, resulting in a limitation in the number of microbial cells that participate in this EET mechanism. The second is EET via self-secreted, naturally-occurring and/or artificially-supplemented soluble electron shuttles (28, 40, 54, 56). In this mechanism, microbial cells are able to perform long-range ET without the necessity of direct contact to solids, whereas their ET is limited by the diffusion of electron shuttles (51). The third is EET via conductive biofilms that are synthesized by microorganisms themselves. The molecular mechanisms conferring conductivity on biological matrices have been tentatively studied on a DMRB, *Geobacter sulfurreducens*, and it has been speculated that extracellular *c*-Cyts (30, 37, 46), extracellular polysaccharides (48), and conductive pili (3, 6, 27, 42, 43) are essential for the functions. EET based on conductive biofilms is considered to be the most efficient among the three mechanisms (51), since a large number of microbial cells in thick biofilms are able to respire distant solid electron acceptors without diffusion resistance (4, 43). Different DMRB may utilize different EET mechanisms, while each may also be able to utilize different mechanisms depending on environmental settings.

We have recently proposed another possibility for long-range ET, which is mediated by microbe/mineral conductive networks. In the presence of (semi)conductive iron-oxide nanoparticles, *Shewanella* strains formed microbe/mineral conductive networks and performed efficient EET (34). It was also demonstrated that microbe/mineral conductive networks were generated by soil microbes, in which *Geobacter* spp. (*e.g.*, those closely related to *Geobacter bremensis* and *Geobacter pelophilus*) grew abundantly (14). Since (semi)conductive iron-oxide minerals are abundantly present in the natural environment (47, 57) and often biologically produced by DMRB themselves (2, 5, 24, 53), such EET is considered to largely contribute to the establishment and functioning of microbial communities. In addition, it has been found that *Geobacter* strains utilize (semi)conductive iron oxides for interspecies electron transfer, facilitating “electric syntrophy” with a nitrate reducer (16) or methanogenic archaea (15). These studies provide new insights into the ecological roles of *Geobacter* spp., whereas information is limited as to how (semi)conductive iron oxides affect the physiology of *Geobacter* populations.

The present study investigated respiratory interactions of *Geobacter* spp. with iron-oxide minerals in axenic cultures. We analyzed five *Geobacter* spp. (listed in Table 1) for their abilities to form microbe/mineral conductive networks. Furthermore, microscopic, electrochemical and transcriptomic analyses of *G. sulfurreducens* were performed to clarify the EET paths used in the absence or presence of (semi)conductive iron oxides, since this was the only strain that formed two different types of EET paths, *i.e.*, conductive biofilm and microbe/mineral network.

## Materials and Methods

### Bacterial strains and culture conditions

*G. sulfurreducens* (DSM12127^T^), *Geobacter metallireducens* (DSM7210^T^), *G. bremensis* (DSM12179^T^), *Geobacter bemidjensis* (DSM16622^T^) and *G. pelophilus* (DSM12255^T^) were obtained from the Deutsche Sammlung von Mikroorganismen und Zellkukturen GmbH (Braunschweig, Germany). The *Geobacter* strains were routinely cultivated in PSN medium with acetate (10 mM) and fumarate (40 mM) as the electron donor and acceptor, respectively, with the exception of nitrate (20 mM) as an electron acceptor for *G. metallireducens*. PSN medium is composed of 10 mM NH_4_Cl, 1 mM KH_2_PO_4_, 0.1 mM MgCl_2_, 0.1 mM CaCl_2_, 0.04 mM MgSO_4_, 20 mM NaHCO_3_, 20 mM 4-(2-hydroxyethyl)-1-piperazineethanesulfonic acid (HEPES), 0.01% Bacto yeast extract, and 1 mL L^−1^ each of vitamin solution, trace element solution and Se/W solution (9). Cultivation was conducted at 30°C under an atmosphere of (N_2_/CO_2_ 80:20 [v/v]) without shaking. Ferrihydrite was prepared as described elsewhere (22). Concentrations of HCl-extractable Fe(II) were determined by the ferrozine method as described elsewhere (23).

### Electrochemical cultivation

Electrochemical cultivation was conducted as described previously (14). A tin-doped In_2_O_3_ (ITO) glass electrode, an Ag/AgCl (KCl sat.) electrode and a platinum wire were used as working, reference and counter electrodes, respectively. After sterilization, the electrochemical cell was filled with the sterilized PSN medium without the vitamin solution. A sterilized, oxygen-free 1 M stock solution of sodium acetate was injected into the cell to give a final concentration of 10 mM. Nanoparticles of hematite and magnetite were synthesized according to previously described methods (11, 32), and injected into the cell with a final concentration of 10 mM as Fe atoms. After removing oxygen from the medium by bubbling with N_2_/CO_2_ gas, *Geobacter* cells grown as described above up to an early stationary phase (optical density at 600 nm [OD_600_] of approximately 0.3) were inoculated to give a final OD_600_ of 0.01. The working electrode was set at +0.2 V (vs. Ag/AgCl) throughout incubation using a HA-1510 potentiostat (Hokuto Denko), and the current was recorded. Cyclic voltammetry (CV) analysis was conducted using the potentiostat HSV-110 (Hokuto Denko) at a scan rate of 10 mV s^−1^. Photo-irradiation was conducted using a xenon lamp SX-UI501XQ (Ushio) as described previously (34). A cut-off (wavelength of >420 nm) glass filter was used to remove UV light from the light source.

### Scanning electron microscopy (SEM) analysis

*G. sulfurreducens* cells precipitated on the ITO electrode were gently washed with HEPES buffer (0.1 M of HEPES, pH 7) and fixed with 2.5% glutaraldehyde in HEPES buffer for 3 hours. Fixed cells were washed three times with HEPES buffer, dehydrated using a graded series of ethanol solutions (30, 50, 75, 90, 95% [v/v]), and dried with a freeze-drying device VFD-21S (Vacuum device) according to the manufacturer’s instructions. The dried samples were coated with platinum and imaged using a VE-9800 microscope (Keyence).

### Microarray analysis

Bacterial cells were collected from precipitates on an ITO electrode, when current density reached approximately 70% of the maximum. Total RNA was isolated using TRIZOL reagent (Invitrogen) according to the manufacturer’s instructions. The quality of RNA was evaluated using an Agilent 2100 Bioanalyzer with RNA 6000 Pico reagents and RNA Pico Chips (Agilent Technologies) according to the manufacturer’s instructions. Specific oligonucleotides (60 mer) were designed for 3,402 genes (corresponding to 99.2% of the total predicted genes in the annotated genome of *G. sulfurreducens* [31]) by the eArray protocol (Agilent Technologies) and fabricated on slide glasses by SurePrint technology (Agilent Technologies). For each gene, 4 spots (with 4 different specific sequences) were printed in one array. A mean value of normalized signal intensities of 4 spots was used for expression analyses. Fluorescence labeling of cDNA, hybridization and scanning of a hybridized array were performed according to the method of two-color microarray-based gene-expression analysis as described previously (12, 13). To minimize dye biases, dyes (Cy3 and Cy5) were swapped for each replicate. RNA isolated from *G. sulfurreducens* cells under non-Fe conditions was used as the reference sample. Fluorescence-dye labeled cDNA for either +Hematite or +Magnetite cultures was mixed with oppositely labeled cDNA as the reference and hybridized with the probes on a microarray. RNA samples from three independent cultures were analyzed for each condition as biological replicates. Microarray data analysis was conducted by the methods described previously (12, 13) using GeneSpring GX ver. 10 (Agilent Technologies). The transcriptome data have been deposited in the CIBEX (Center for Information Biology gene EXpression) database under accession number CBX206.

### Quantitative reverse transcription PCR (qRT-PCR) analysis

PCR primers used for qRT-PCR were designed by Nihon Gene Research Laboratories, and listed in Table S1. Quantitative gene expression analysis was performed by real-time RT-PCR using a LightCycler system and a LightCycler RNA Master SYBR Green I kit (Roche Applied Science) as described previously (12). Data of qRT-PCR analysis of the 16S rRNA gene were used as a reference for normalization of the gene expression values.

## Results and Discussion

### EET by *Geobacter* spp

Five *Geobacter* strains listed in Table 1 were grown in anaerobic bottles to compare their EET properties. *G. sulfurreducens* and *G. metallireducens* are members of the *G. metallireducens* clade in the family *Geobacteraceae* and have been extensively studied for their physiology and genetics as model DMRBs (1, 26, 31). *G. bremensis* and *G. bemidjensis* are affiliated with subsurface clade 1 in the family *Geobacteraceae*, while *G. pelophilus* is affiliated with the subsurface clade 2 (36, 50). While *Geobacter* spp. in the subsurface clades have often been detected as major members of various natural communities and enrichment cultures (8, 14), only limited information is available for their physiology.

**Fig. 1:**
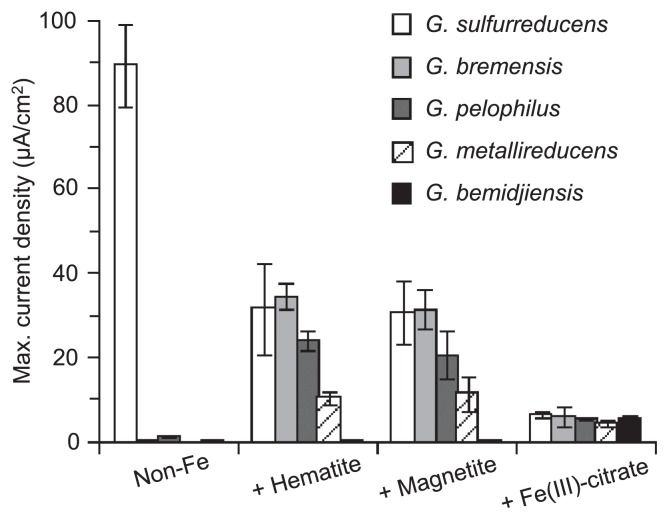
Current generation by the five *Geobacter* strains in electrochemical cells in the absence and presence of iron-oxide minerals. Maximum current densities were determined from current versus time curves shown in Fig. S2. Data are presented as the means of three independent cultures, and error bars represent standard deviations.

As the initial step in our investigation, their abilities to reduce insoluble, poorly crystalline iron oxides (ferrihydrite) were checked (Fig. S1). All reduced ferrihydrite as reported (19), confirming that they have abilities for EET. Next, in order to examine EET to electrodes (*i.e.*, electric current generation), each strain was cultivated in an electrochemical cell with acetate (10 mM) as the sole electron donor and an ITO electrode (poised at +0.2 V vs. Ag/AgCl) as the sole electron acceptor. Maximum current densities were determined from current versus time curves (Fig. S2) and are summarized in Fig. 1. Under this condition (non-Fe control), *G. sulfurreducens* achieved a large current density (up to 90 μA cm^−2^), while the others showed much lower current values (0.4 to 1.2 μA cm^−2^). The ability of *G. sulfurreducens* to generate such a large current has frequently been reported and appears to be conferred by the production of thick conductive biofilm (27, 37, 43).

Supplementation with 10 mM ferric ion (chelated with citrate) affected current generation by the *Geobacter* strains (+Fe(III)-citrate samples in Fig. 1). Different from the results of non-Fe control cultures, the five *Geobacter* strains achieved similar current densities (4.6 to 6.6 μA cm^−2^). Under this condition, ferric/ferrous ions could work as electron shuttles; *Geobacter* reduces Fe^3+^ to Fe^2+^, Fe^2+^ diffuses to electrode surfaces and is oxidized back to Fe^3+^ by releasing electrons, resulting in the current generation. It is noteworthy that supplementation with Fe ions resulted in a marked decrease in current density in the *G. sulfurreducens* culture, implying that Fe ions suppressed the production of biological conductive matrices by *G. sulfurreducens*. This result is consistent with previous findings; *G. sulfurreducens* biofilms grown on electrodes in the presence of a soluble electron acceptor (*e.g.*, fumarate) showed neither active current generation nor electrical conductance (27, 37).

### Effects of (semi)conductive iron oxides on EET by *Geobacter* spp

Electrochemical cultures were supplemented with either hematite (semiconductor) or magnetite (conductor) nanoparticles (10 mM as Fe atom) (+Hematite or +Magnetite in Fig. 1). Supplementation with hematite or magnetite similarly affected current production by *G. bremensis*, *G. pelophilus* and *G. metallireducens*. These *Geobacter* strains generated larger currents in the (semi)conductive mineral-amended cultures (11 to 35 μA cm^−2^) than in the non-Fe and +Fe(III)-citrate cultures. The results indicate that these *Geobacter* strains are able to construct microbe/mineral conductive networks as observed in *Shewanella* spp. (34). In contrast, current generation by *G. bemidjensis* was not affected by supplementation with the (semi)conductive minerals, suggesting that the ability to construct microbe/mineral conductive networks is not a universal feature in the genus *Geobacter*.

In the case of *G. sulfurreducens*, current densities in hematite- or magnetite-supplemented cultures were comparable to those in *G. bremensis* and *G. pelophilus* cultures, while current densities were smaller than in the non-Fe *G. sulfurreducens* culture (Fig. 1). Two plausible explanations were possible for these results, namely, (1) *G. sulfurreducens* produced conductive biofilm matrices, while iron-oxide nanoparticles inhibited long-range ET via conductive biofilms; (2) *G. sulfurreducens* generated currents via microbe/mineral conductive networks without producing conductive biofilms.

### EET paths of *G. sulfurreducens*

**Fig. 2:**
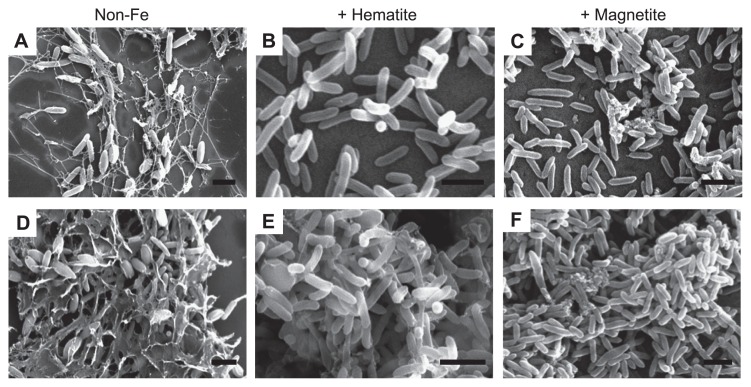
SEM images of *G. sulfurreducens* cells growing on ITO electrodes. Cells were sampled during current generation from non-Fe (A, D), +Hematite (B, E), and +Magnetite (C, F) cultures. Images show cells when current was increased (A–C) and those after current densities reached the maximum values (D–F). Bars are 2 μm.

In order to compare EET paths of *G. sulfurreducens* in the presence and absence of iron-oxide nanoparticles, microscopic and (photo-)electrochemical analyses were conducted for *G. sulfurreducens* cells grown on the electrodes. It has been reported that *G. sulfurreducens* produces thick biofilm structures on electrode surfaces under current-generating conditions (39, 43), and such biofilms have been observed with SEM (10, 48). During the preparation of SEM samples, iron-oxide nanoparticles that were precipitated and loosely attached to cell surfaces were removed. In the non-Fe sample, *G. sulfurreducens* produced thick biofilms, in which cells were surrounded by extracellular polymeric substances (EPSs) (Fig. 2A and D), as has been observed in previous studies (10, 48). It has been shown that EPSs are crucial for long-range ET in conductive biofilms, in which they function as nets to trap extracellular *c*-Cyts (48). In contrast, EPS-like matrices were not observed around *G. sulfurreducens* cells when they grew in the presence of hematite (Fig. 2B and E) or magnetite (Fig. 2C and F). These observations indicate that the production of biological conductive matrices was suppressed by supplementation of hematite or magnetite. This may be an important physiological response for *G. sulfurreducens* to proliferate on solid electron acceptors, and future studies will direct qualitative and quantitative identification of this response.

**Fig. 3:**
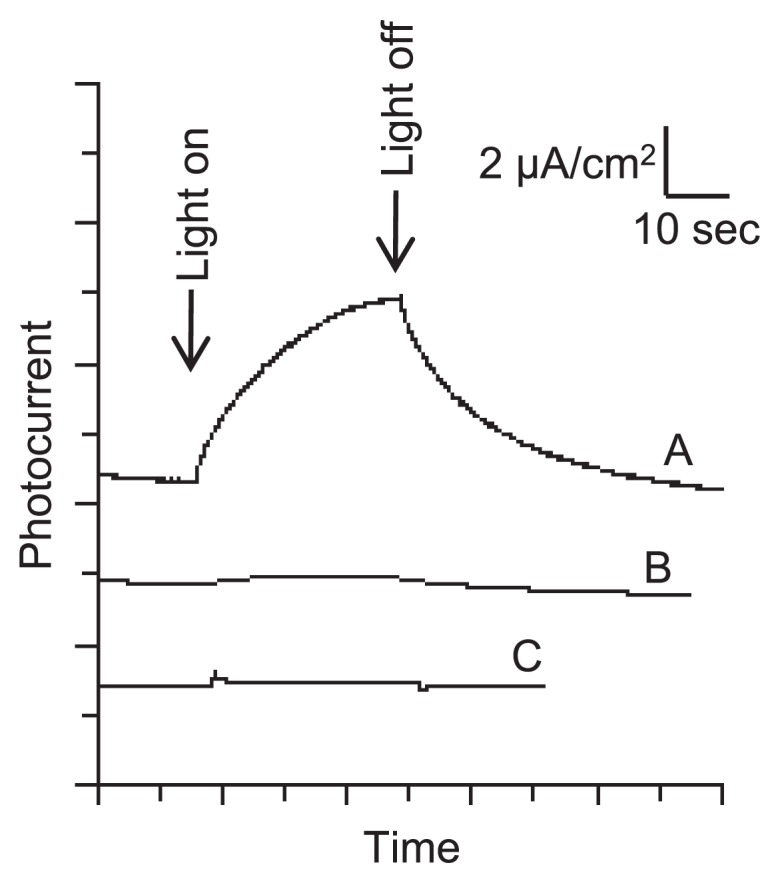
Photo-induced currents from hematite-amended *G. sulfurreducens* cultures. Light (>420 nm) was irradiated for 30 sec at the time points indicated by arrows. Trace A, *G. sulfurreducens* cells in the presence of hematite nanoparticles; B, a control without *G. sulfurreducens* cells; C, a control without hematite nanoparticles.

A photo-electrochemical technique was used to confirm the construction of a microbe/mineral conductive network by *G. sulfurreducens*. Photo-irradiation of semiconductive iron oxides is known to induce the excitation of electrons from the valence band to the conduction band and generate a current (photo-current) when microbe/mineral conductive networks are formed on electrodes (34). We performed this analysis and found that the *G. sulfurreducens*/hematite mixture also generated photo-current (Trace A in Fig. 3), indicating that microbe/mineral conductive networks were formed by *G. sulfurreducens*. Photo-current densities reached 4 to 5 μA cm^−2^, which is comparable to those from *Shewanella*/hematite biofilms formed in similar experimental settings (34). Control samples without *G. sulfurreducens* cells or hematite nanoparticles (Trace B or C in Fig. 3, respectively) generated low photo-current (<0.2 μA cm^−2^), suggesting that both bacterial cells and hematite nanoparticles are necessary for conductive networks.

**Fig. 4:**
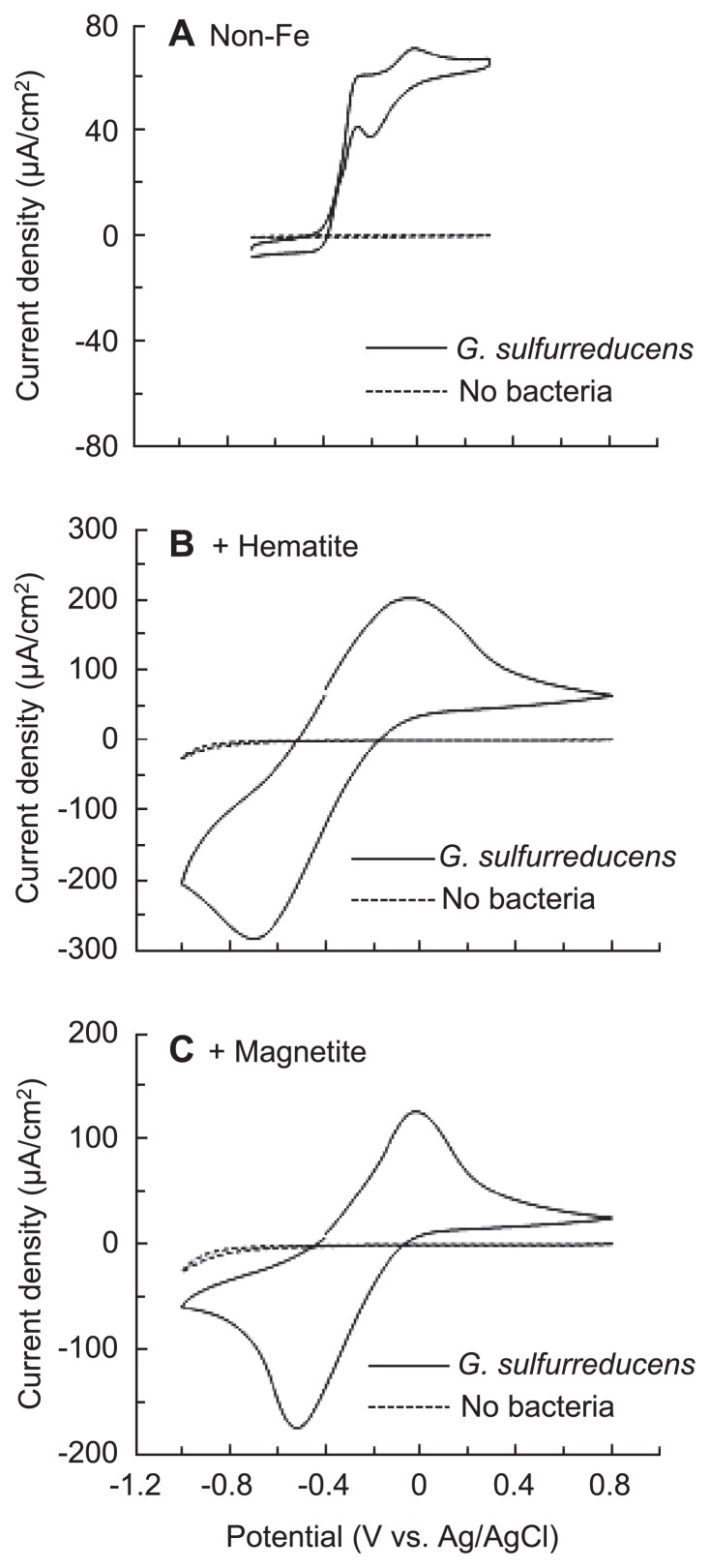
CVs of *G. sulfurreducens* electrochemical cultures (solid lines) in the absence of iron oxides (A), in the presence of hematite (B), and in the presence of magnetite (C). CVs for control samples (without *G. sulfurreducens* cells) are also presented (broken lines).

EET paths of *G. sulfurreducens* were also analyzed by CV under current-generating conditions (Fig. 4). *G. sulfurreducens* biofilm formed under non-Fe conditions showed a sigmoidal CV curve (Fig. 4A) typical of conductive biofilm of this strain (29, 30, 46). Supplementation with hematite and magnetite markedly altered its CV pattern (Fig. 4B and 4C, respectively); symmetric redox couples appeared and peak currents were largely increased. Similar symmetric CV patterns have been reported for *Shewanella* cultures amended with hematite nanoparticles (34); in that report, the redox peaks were attributed to OM *c*-Cyts that were interconnected via hematite nanoparticles. We therefore consider that the CV data shown in Fig. 4B and 4C support the idea that microbe/mineral conductive networks (rather than conductive biofilms) were formed in the presence of hematite and magnetite nanoparticles. Similar symmetric CV patterns were also observed for *G. bremensis* (Fig. S3), a representative of *Geobacter* strains that do not form conductive biofilms.

The microscopic, photo-electrochemical and CV data consistently suggested that, when iron-oxide nanoparticles were present, *G. sulfurreducens* utilized microbe/mineral conductive networks for EET as a substitute for conductive biofilms. The ability to utilize microbe/mineral networks was also found in other *Geobacter* strains, including *G. bremensis*, *G. pelophilus* and *G. metallireducens*. Among them, *G. bremensis* is a member of subsurface clade 1, whose closely related sequences have been detected abundantly from various anaerobic ecosystems (8, 14), suggesting that these *Geobacter* populations occurred in the environment using microbe/mineral conductive networks.

### Effects of (semi)conductive iron oxides on gene expression in *G. sulfurreducens*

The above experiments showed the morphological and electrochemical differences in *G. sulfurreducens* EET paths in the absence and presence of (semi)conductive iron oxides (*i.e.*, conductive biofilm and microbe/mineral network, respectively). In order to gain insights into the molecular mechanisms underlying these differences in its EET path, gene-expression analyses were performed for current-generating *G. sulfurreducens* cells in the absence and presence of iron oxides. To date, studies have been performed to identify molecular components that constitute the conductive biofilm of *G. sulfurreducens* (20, 21, 27, 37, 48), while no information has been obtained for the microbe/mineral networks. It has been suggested that many *c*-Cyts play crucial roles in EET; for instance, electrons are transferred from inner-membrane (IM) *c*-Cyts to OM *c*-Cyt complexes (*e.g.*, OmcB and OmcS) via soluble periplasmic *c*-Cyts (*e.g.*, PpcA). Electrons are further transferred to extracellular electron acceptors directly from the OM *c*-Cyts or via conductive biofilm. It is speculated that the conductivity of the biofilm is conferred by extracellular *c*-Cyts (*e.g.*, OmcZ, OmcB and OmcS) and conductive pili (27, 30, 37, 43, 46). In the present study, we conducted microarray analyses to investigate differential gene expression in *G. sulfurreducens* grown under non-Fe, +Hematite and +Magnetite conditions. The microarray system for *G. sulfurreducens* was customized, and was validated by comparing expression-fold data for several genes obtained by the microarray system and those obtained by qRT-PCR (Fig. S4).

**Fig. 5:**
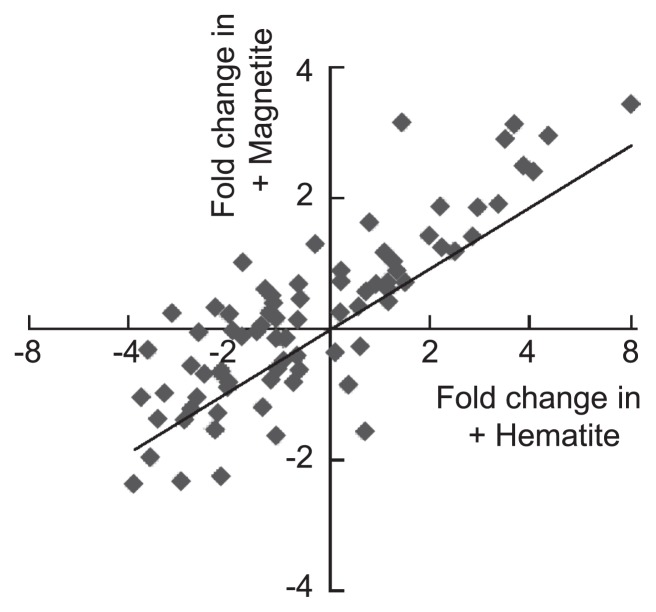
Expression profiles of 82 *c*-Cyt genes in the *G. sulfurreducens* genome. Expression fold changes in hematite- and magnetite-amended conditions against the control (non-Fe) were calculated from the microarray data. An approximation curve (y=0.50x + 0.20, *r*=0.84) derived from the least-square method is shown.

Microarray analysis revealed that the expression of genes for IM and periplasmic *c*-Cyts (*macA* and *ppcA*) was not significantly different between non-Fe and (semi)conductive mineral-amended conditions (data not shown). In addition, genes for OM and extracellular *c*-Cyts (*omcB*, *omcS* and *omcZ*) and a gene for the main component of pili (*pilA*) (42) were mostly down-regulated in the (semi)conductive mineral-amended samples (Table 2). In contrast, we also found that some *c*-Cyt genes were up-regulated in the presence of iron oxides (Table 2). Comparisons of the expression patterns of *c*-Cyt genes in the *G. sulfurreducens* genome under +Hematite and +Magnetite conditions are presented in Fig. 5, showing that they were correlated well. Among the over 100 *c*-Cyt genes present in the *G. sulfurreducens* genome (31), twelve *c*-Cyt genes were found to be highly expressed under +Hematite and +Magnetite conditions (Table 2). These *c*-Cyts are likely ET components constituting microbe/mineral conductive networks, although these *c*-Cyts (except for PgcA [52]) have not yet been analyzed in molecular and physiological studies. It is also noteworthy that 7 *c*-Cyts are encoded in a gene cluster (GSU3216–3233) which contains two sets of two-component regulatory systems (GSU3216–3217 and GSU3229–3230). The genes for these 7 *c*-Cyts have typical signal-peptide sequences, supporting the idea that they are components of EET paths in microbe/mineral conductive networks. It is therefore suggested that *c*-Cyts used in the conductive biofilm and in the microbe/mineral network are different. Further molecular and physiological analyses are necessary to elucidate their exact roles in conductive networks. In addition, comparative genomics for the five *Geobacter* strains may provide fruitful information as to the c-Cyts necessary for microbe/mineral networks, for which it is desirable to determine genome sequences of *G. pelophilus* and *G. bremensis*.

### Implications

The present study demonstrates that *Geobacter* strains have the ability to construct microbe/mineral conductive networks in the presence of (semi)conductive iron-oxide nanoparticles. In addition to this ability, *G. sulfurreducens* has the unique ability to construct conductive biofilms, and this bacterium utilizes either of these long-range ET paths depending on the environmental setting. It is suggested that microbe/mineral conductive networks are widely used by bacteria in the genus *Geobacter*, while only limited members are able to form conductive biofilms.

When iron-oxide nanoparticles were present, *G. sulfurreducens* generated microbe/mineral networks for electrode respiration, even though the current density achieved with the microbe/mineral networks was much less than that with conductive biofilms (Fig. 1). This result may be relevant to our previous findings; in the absence of iron-oxide nanoparticles, electrochemical cultivation enriched bacteria closely related to *G. sulfurreducens* from rice paddy-field soil, while supplementation of the soil with iron oxides allowed *Geobacteraceae* populations affiliated with the subsurface clades to occur abundantly (14). These results indicate that the microbe/mineral network has ecological advantages over conductive biofilm. We propose that this finding can be explained by considering the cost of constructing long-range ET paths; namely, large amounts of EPSs and extracellular proteins (*e.g.*, *c*-Cyts and pili) are necessary for constructing conductive biofilms, and *Geobacter* may consume substantial amounts of carbon and energy sources for this purpose. The use of environmental costless materials (*e.g.*, conductive minerals) therefore confers ecological advantages upon *Geobacter* populations, resulting in the preferential use of these materials and the outgrowth of bacteria using these materials. It is likely that the conductive biofilm is the last resort of *G. sulfurreducens* and is synthesized only when there is no easily accessible electron acceptor.

It has recently been argued that electric currents play important roles in biogeochemical reactions in the environment (15, 35, 41, 44). The present study also supports this idea by showing that *Geobacter* strains preferentially use (semi)conductive minerals for their EET. Further investigation will shed light on the impact of (semi)conductive minerals on microbial physiology and evolution in natural ecosystems.

## Supplementary Material



## Figures and Tables

**Table 1:** *Geobacter* spp. used in this study

Strain name	Clade in the family *Geobacteraceae*	Ferrihydrite reduction	Ability for EET^a^	

			Non-Fe	+ Fe oxides
*G. sulfurreducens*	*G. metallireducens*	+	+	+
*G. metallireducens*	*G. metallireducens*	+	−	+
*G. bremensis*	Subsurface 1	+	−	+
*G. bemidjensis*	Subsurface 1	+	−	−
*G. pelophilus*	Subsurface 2	+	−	+

a Current production (>10 μA cm^−2^) was observed or not (see Fig. 1).

**Table 2:** Differentially expressed *c*-Cyt and related genes in +Hematite and +Magnetite cultures

Gene ID	Annotation (gene name)	+Hematite		+Magnetite	
	
		Fold change^a^	*p* value	Fold change^a^	*p* value
Up-regulated in + Hematite and +Magnetite					
GSU0701	cytochrome c family protein (OmcJ)	4.08	2E-4	2.13	4E-3
GSU1771	cytochrome c family protein (PgcA)	2.17	6E-6	1.55	1E-3
GSU2203	cytochrome c family protein (OmcK)	2.68	1E-3	1.65	5E-3
GSU2204	cytochrome c family protein, putative	2.36	2E-3	1.53	2E-3
GSU2299	cytochrome c family protein	3.20	7E-6	1.96	1E-5
GSU3218	cytochrome c family protein	4.51	8E-4	2.81	1E-3
GSU3221	cytochrome c family protein	3.37	4E-5	2.77	2E-4
GSU3223	cytochrome c family protein	3.82	4E-5	2.40	8E-4
GSU3226	cytochrome c family protein	2.80	7E-4	1.92	4E-3
GSU3228	cytochrome c family protein	8.09	8E-6	3.33	5E-4
GSU3232	cytochrome c family protein	3.58	3E-4	2.98	9E-4
GSU3233	cytochrome c family protein	2.15	2E-4	1.93	7E-3
Down-regulated in +Hematite and +Magnetite					
GSU0274	cytochrome c family protein	−3.91	3E-5	−2.28	4E-5
GSU0592	cytochrome c family protein (OmcQ)	−3.30	8E-5	−1.60	5E-5
GSU2737	cytochrome c family protein (OmcB)	−2.99	2E-4	−1.09^b^	2E-1
GSU0670	cytochrome c family protein (OmcX)	−2.63	2E-3	−1.52	4E-3
GSU1760	Cyd-5, cytochrome c3 (PpcE)	−2.20	3E-5	−1.70	4E-3
GSU1996	cytochrome c family protein	−3.48	1E-6	−1.97	2E-4
GSU2076	cytochrome c family protein (OmcZ)	−2.15	1E-3	−2.18	3E-3
GSU2201	cytochrome c family protein	−2.25	6E-5	−1.70	5E-3
GSU3259	cytochrome c family protein	−2.82	3E-4	−2.23	2E-3
GSU1496	pilin domain-containing protein (PilA)	−1.68	7E-3	−2.18	3E-3
GSU2504	cytochrome c family protein (OmcS)	−1.45^b^	1E-1	−1.76	7E-4

a Gene expressions under +Hematite and +Magnetite conditions are expressed as positive (up-regulated) or negative (down-regulated) fold changes against their expressions under non-Fe condition.

b Not statistically significant.
