# Supplementary material for: Iron-Oxide Minerals Affect Extracellular Electron-Transfer Paths of Geobacter spp
Source: Microbes Environ. 2013 Jan 30;28(1):141–8. doi: 10.1264/jsme2.ME12161 (PMC4070692; doi:10.1264/jsme2.ME12161)
Supplement: Supplementary file 1 [file 28_141_s1.pdf]

Table S1. qRT-PCR primers used in this study.

Primer name	Target gene	Sequence (5' to 3')	Reference
Geo494F	16S rRNA	AGG AAG CAC CGG CTA ACT CC	Holmes et al., 2002
Geo825R	16S rRNA	TAC CCG CRA CAC CTA GT	Holmes et al., 2002
omcS-1150F	omcS	GGC AAC TCG ATC TAT GGT A	This study
omcS-1242R	omcS	GGT AAG GAG CAA ACT TGT CA	This study
omcZ-1049F	omcZ	AAA GCG AGA TCA AGG TGT CC	This study
omcZ-1193R	omcZ	GCC GGT AAT GGT AAG GGT CT	This study
pilA-51F	pilA	GAC ACT TAT GCT TCA GAA ACT CAG	This study
pilA-166R	pilA	TTG TAC GCC TTG ACA CGA TA	This study

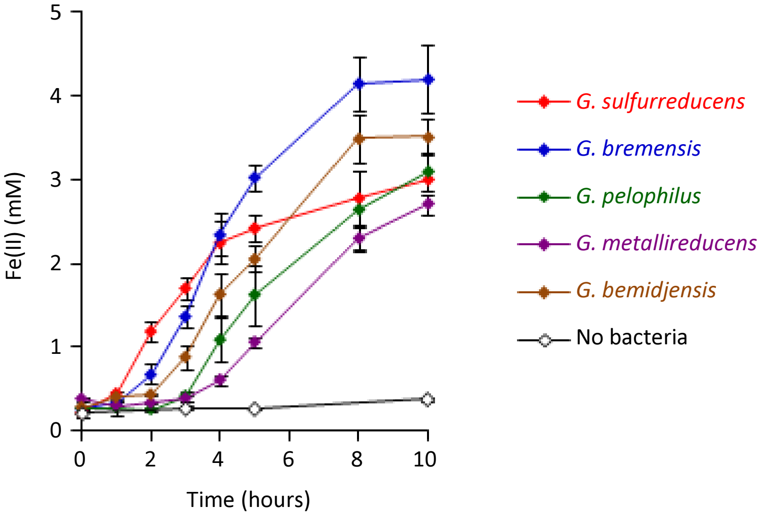

Fig. S1. Ferrihydrite reduction by *Geobacter* species

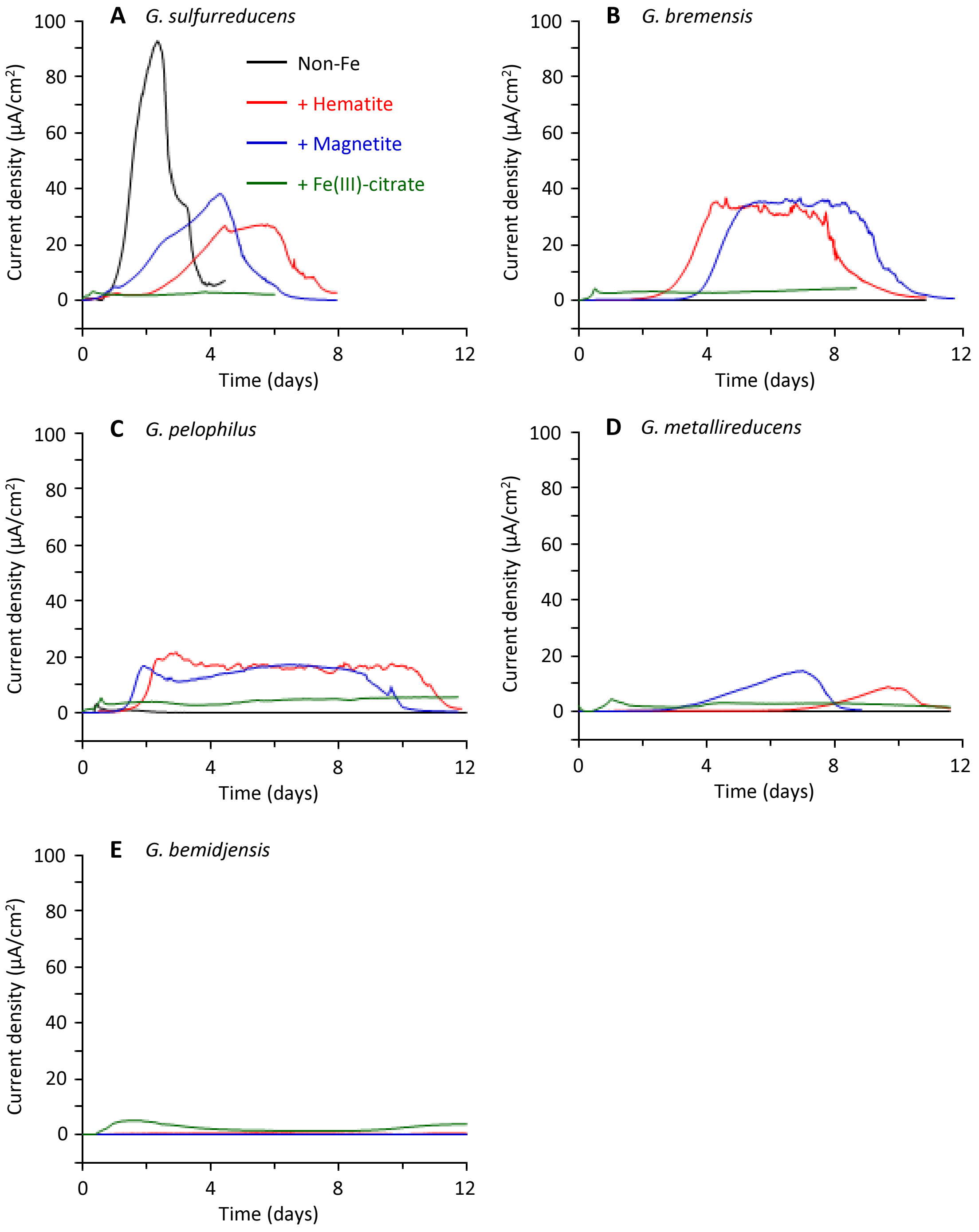

Fig. S2. IT curves for the five *Geobacter* strains in the absence and prsence of iron species.

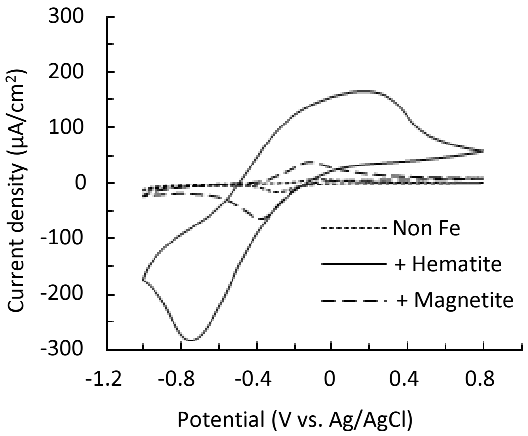

Fig. S3. CV patterns of *G. bremensis.*

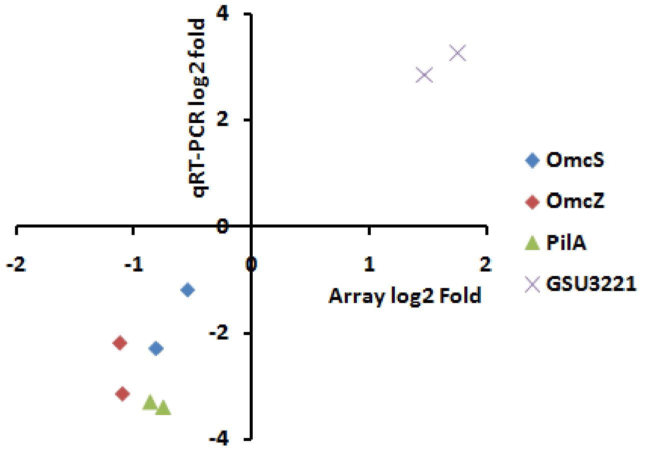

Fig. S4. Comparison in expression-fold data between microarray and qRT-PCR
